# The Generation and Identity of Human Myeloid-Derived Suppressor Cells

**DOI:** 10.3389/fonc.2020.00109

**Published:** 2020-02-07

**Authors:** Caroline Bergenfelz, Karin Leandersson

**Affiliations:** ^1^Department of Translational Medicine, Division of Experimental Infection Medicine, Lund University, Malmö, Sweden; ^2^Department of Translational Medicine, Cancer Immunology, Lund University, Skåne University Hospital, Malmö, Sweden

**Keywords:** myeloid-derived suppressor cell, cancer, infection, development, differentiation, maturation, activation, tolerance

## Abstract

Myeloid-derived suppressor cells (MDSCs) are cells of myeloid lineage with a potent immunosuppressive capacity. They are present in cancer patients as well as in patients with severe inflammatory conditions and infections. MDSCs exist as two main subtypes, the granulocytic (G-MDSCs) and the monocytic (Mo-MDSCs) type, as defined by their surface phenotype and functions. While the functions of MDSCs have been investigated in depth, the origin of human MDSCs is less characterized and even controversial. In this review, we recapitulate theories on how MDSCs are generated in mice, and whether this knowledge is translatable into human MDSC biology, as well as on problems of defining MDSCs by their immature cell surface phenotype in relation to the plasticity of myeloid cells. Finally, the challenge of pharmacological targeting of MDSCs in the future is envisioned.

## A Brief History of Myeloid-Derived Suppressor Cells (MDSCs)

Already in 1929, cancer was found to be associated with an aberrant myelopoiesis (1). In the late 1960–80s, experiments revealed leukocytosis, granulocytosis, and extramedullary myelopoiesis in tumor-bearing mice (2–5). This aberrant emergency myelopoiesis was driven by tumor cell-derived colony stimulating factors GM-CSF, G-CSF and M-CSF (5–10), that also promoted cancer cell growth (8). During the same time period, the “left shift” test was established as a clinical test in patients with severe bacterial infections. “Left shift” is defined as an increased ratio of immature myelocytes, metamyelocytes and band neutrophils (i.e., shifted to the left of the differentiation model) in blood smears from patients (11–14). A similar “left shift” is also proposed in patients with sterile inflammation and cancer, although not necessarily associated with as severe leukocytosis (15, 16). The leukocytosis in sepsis patients is a normal feedback regulation to replace consumed neutrophils, and is likely caused by similar factors that cause the aberrant myelopoiesis in cancer, including colony stimulating factors, other growth factors and secondary host responses such as damage associated molecular patterns (DAMPs) (16). The first studies showing that the increased systemic immature myeloid cells in tumor-bearing mice where immunosuppressive (“natural suppressor cells”), came in the late 1970s (3, 17–19) but not until 1996 this was first shown in humans (20). Over the following years, the definition of subpopulations and mechanisms of action were heavily investigated (21) and the consensus terminology myeloid-derived suppressor cells (MDSCs) was established in 2007 (22). Today, MDSCs are divided into two main subtypes; the granulocytic-MDSCs [G-MDSCs or polymorphonuclear (PMN)-MDSCs] and monocytic-MDSCs (Mo-MDSCs). A third subpopulation has also been proposed, the early-stage MDSC (eMDSC) that lacks both CD14 and CD15 expression, which will not be covered in this review (23). All subpopulations above are excellently reviewed from different angels in previous publications (15, 16, 21).

## Defining Myeloid-Derived Suppressor Cells (MDSCs) in Mice and Men

The current definition of MDSCs is that that they should be of myeloid origin and enriched in mice/patients with cancer or severe disease, display an immature surface phenotype and with the key defining trait being their potent immunosuppressive capacity (23–26). Using these criteria, they are further divided into Mo-MDSCs and G-MDSCs (26–28). In this review we will use the nomenclature G-MDSC, and not PMN-MDSCs, since this latter population consists of cells with heterogenous morphology and not only polymorphonuclear cells (29, 30). In mice Mo-MDSCs are defined by the surface phenotype CD11b^+^Ly6G^−^Ly6C^hi^ and G-MDSCs by CD11b^+^Ly6G^+^Ly6C^lo^ (31). In humans Mo-MDSCs are CD14^+^HLA-DR^−/lo^ and G-MDSCs CD11b^+^CD15^+^CD14^−^CD33^+/lo^CD66b^+^ cells with a low density [low density granulocytes (LDGs)] (23, 32, 33), and are hence present in the peripheral blood mononuclear (PBMC) fraction of gradient centrifugations. Many markers are still appearing in efforts to further define the human MDSC subsets (34), one being S100A9 (35, 36).

**Figure 1:**
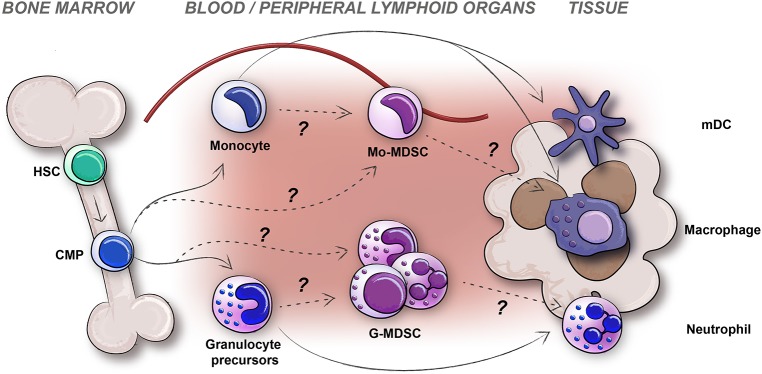
The generation, distribution, and plasticity of human MDSCs subtypes; monocytic-MDSCs (Mo-MDSC) and granulocytic-MDSCs (G-MDSCs) are pictured. Differentiation of myeloid cells in healthy individuals (solid arrows) and potential origin of MDSCs during disease (dashed arrows) are indicated.

Using these criteria, MDSCs have been studied successfully in mice for many years, and in humans for slightly more than a decade with varying results. In mice, CD11b^+^Ly6G^−^Ly6C^hi^ Mo-MDSCs and CD11b^+^Ly6G^+^Ly6C^lo^ G-MDSCs with immunosuppressive capacity can be enriched and studied from peripheral blood, secondary lymphoid organs and tumors, with quite consistent results. In humans, using the Mo-MDSC CD14^+^HLADR^−/lo^ and G-MDSC CD11b^+^CD15^+^CD14^−^CD33^+/lo^CD66b^+^ cell markers for identification has turned out to be complex. There are multiple reasons for this, some being; (i) *Immaturity* vs. *Plasticity*; the problem of defining heterogeneous cell populations using cell surface markers, (ii) *Subpopulations* vs. *Technical issues*; the problem of comparing human blood and tissue MDSCs along with the problem of investigating human MDSCs by other means than flow cytometry of PBMCs as source, (iii) *In vitro* vs. *In vivo*; as recently suggested, functional studies on human cells are for natural reasons more often performed *ex vivo*, but all *in vitro* generated human MDSCs should by all means be defined as “MDSC-like” cells (23). Therefore, questions still remain concerning subsets, origin, and function of human MDSCs. If the debate concerning the true identity of human MDSCs, and subsets thereof, would be of only philosophical character, one could still adhere to the most important notion that they are myeloid cells with an immunosuppressive capacity, and an immature surface phenotype. However, when the question concerns how to be able to target them in cancer patients, the issue of defining human MDSC subsets identity and their origin, is still in need of improvement. Below we will discuss the generation and identity of the different human MDSC subsets and put them in context with their sites of distribution (Figure 1).

## Human Peripheral Blood G-MDSCs

**Figure 2:**
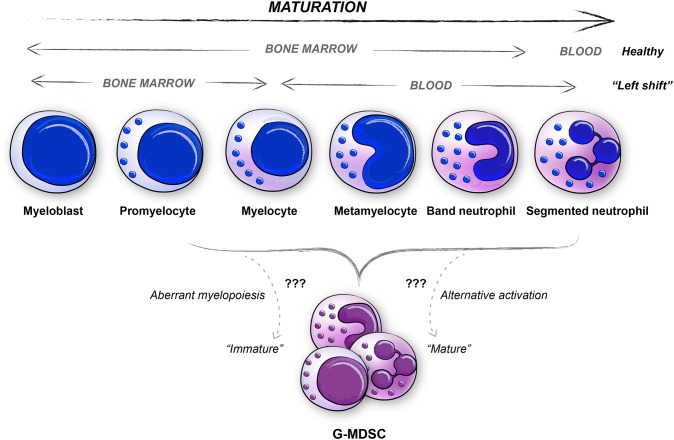
Similarities between neutrophil maturation stages and human G-MDSCs. The various differentiation stages of neutrophils are depicted, both for healthy subjects and for patients with severe diseases as represented by the “Left shift.” Human G-MDSCs subpopulations are represented in most neutrophil maturation stages, ranging from promyelocyte to mature neutrophil (PMN, polymorphonuclear cells).

G-MDSCs are a heterogeneous population of cells of the granulocytic lineage. In mice, the surface marker definition is CD11b^+^Ly6G^+^Ly6C^lo^, while in human the definition is CD11b^+^CD15^+^CD14^−^CD33^+/lo^CD66b^+^ cells with a low density (LDGs) (23, 30). As for all MDSCs, the most critical trait is their immunosuppressive activity. For G-MDSCs, suppression of immune responses is conveyed in an antigen-specific manner, and mediated by secreted factors such as reactive oxygen species (ROS) and G-CSF, and enzymatic mediators like Arginase I (ARG1), although the Arginase function is reported with varying results in humans partly due to inconsistencies in measuring protein levels as compared to enzymatic activity (23, 37, 38). The functional aspects of G-MDSCs, have been excellently reviewed elsewhere and will therefore not be covered in detail here (30, 39). The generation of human G-MDSCs is still debated, mainly since the morphology of human G-MDSCs present a heterogeneous population of cells ranging from immature neutrophils to mature polymorphonuclear (PMN) neutrophils (Figure 2) (29, 32, 38, 40, 41). The “left shift” (11–14), or emergency myelopoiesis exporting immature myeloid granulocytes, may be considered when investigating the morphology and generation of isolated human peripheral blood G-MDSCs (Figure 2). According to previous literature, PMN shaped G-MDSCs (Box 1) can be discriminated from steady-state neutrophils based on a PMN morphology with fewer granules (23). However, in humans, the markers CD11b^+^CD15^+^CD14^−^CD33^+/lo^CD66b^+^ enrich for neutrophils at all maturation stages; from myelocytes to mature neutrophils (Figure 2, Table 1), including cells with fewer granules thus making this distinction difficult (23, 30, 45). Some markers that have been identified to distinguish immature neutrophils from the PMN shaped G-MDSCs are CD10, CD13, CD16, and CD38 which all represent different stages of neutrophil maturation (Table 1), thus supporting that the PMN shaped G-MDSCs are more mature (46–52). However, as discussed below, there are also studies suggesting that immunosuppressive G-MDSCs with an immature surface phenotype and morphology, could derive from de-differentiated or reprogrammed mature neutrophils into immunosuppressive G-MDSCs (29, 53, 54). The traditional view that immunosuppressive *bona fide* G-MDSC are immature cells, is being challenged by current literature indicating that mature cells may also be immunosuppressive. The immature neutrophils (the non-PMN G-MDSCs in Figure 2, Table 1, Box 1), make up ~5–15% of all LDGs in the peripheral blood of cancer patients, probably varying with cancer type and stage (55). Whether the immature neutrophils are more immunosuppressive than the PMN shaped G-MDSCs, thus representing the *bona fide* G-MDSCs, is currently debated (30, 38, 55). There is also a possibility that the immature neutrophils, or subsets thereof, may be mature cells of some other lineage, exemplified by fibrocytes (56). Immature neutrophils are proposed to have a longer half-life and therefore also to survive longer in tissues and tumors, as mentioned below (57). The difference between immature neutrophils and the more mature PMN shaped G-MDSCs regarding function is not clear, but ARG1/iNOS may be mediators preferably used by the immature neutrophil G-MDSCs, as compared to their PMN shaped counterpart (30, 52). Lately, lectin-type oxidized LDL receptor 1 (LOX1) has been suggested as a marker that may identify human G-MDSCs at the functional level (47, 52, 58).

Box 1 **Explanation to nomenclatures used in this review.**

**Immature neutrophil G-MDSCs:** G-MDSCs that are derived from immature neutrophils or G-MDSCs that represent immature neutrophils with non-PMN shaped nuclei and with immunosuppressive activity.

**PMN shaped G-MDSCs:** G-MDSCs that are derived from mature or activated neutrophils or G-MDSCs that represent mature or activated neutrophils with PMN shaped nuclei and with immunosuppressive activity.

**Table 1:** Selected surface phenotypes during neutrophil differentiation, with expression levels of indicated markers in specific neutrophil subsets indicated (30, 42–44).

	**GMP**	**Myeloblast**	**Promyelocyte**	**Myelocyte**	**Metamyelocyte**	**Band cell**	**Neutrophil**	**Activated neutrophil**
CD33	+++	+++	+++	++	+	+	+	+
CD34	+	++	–	–	–	–	–	–
CD10	–	–	–	–	–	–/+	++	+
CD11b	–	–	–/+	+/++	++	++	++	++
CD13	++	+++	+++	+	+/++	++	+++	+++
CD14	–	–	–	–	–	–	–	–/+
CD15	–	+	+++	+++	+++	+++	+++	+++
CD16	–	–	–	–	+/+++	++	+++	++/+++
CD24	–	–	–	++	++	++	++	+++
CD31	–/+	–	–	–	–	–	–/+	+
CD38	++	++	–	–	–	–	–	–
CD62L	++	++	++	++	++	++	++	+
CD64	++	+	+	++	++	–	–	–/+
CD66b	–	–	+++	+++	++	++	++	++

Presently, there is no firm evidence that human PMN shaped G-MDSCs are anything else than activated neutrophils. Mature activated neutrophils may also acquire a low density and thus be isolated in the LDG/PBMC fraction of human peripheral blood (59). Activated neutrophils can be immunosuppressive by inhibiting T cell proliferation via ROS. Neutrophil extracellular traps (NETs) should, however, inevitably induce neutrophil cell death, although with a slight delay (60). Nevertheless, since G-MDSCs theoretically should live longer than activated neutrophils, a unique PMN shaped G-MDSC population has been proposed (23, 61). There are contradicting findings available from gene expression profiles of isolated cancer patient derived G-MDSCs, concerning whether G-MDSCs are activated neutrophils or unique G-MDSC cell populations, a fact that mirrors the complexity of investigating this heterogeneous population of cells in different indications. Indeed, the isolation procedure and choice of neutrophil source, as well as the inter- and intra-patient variation in numbers of immature neutrophils as compared to PMN shaped G-MDSCs, will unequivocally lead to unique profiles for each study (47, 62). Newly introduced methods like multiparameter, multidimensional imaging, single cell RNA Sequencing and mass cytometry by time of flight (CyTOF), will hopefully lead to a better understanding of the heterogeneity of G-MDSCs and their unique subtypes. Indeed, using multidimensional imaging, LOX1^+^ G-MDSCs were recently found to co-express the neutrophil activation marker MPO (58, 63).

The distinctive function of PMN shaped G-MDSCs should be debated. Even though PMN shaped G-MDSCs are immunosuppressive, they could still be classified as conventional activated neutrophils, or as neutrophils with an alternative activation. Indeed, a high Neutrophil to Lymphocyte Ratio (NLR) in cancer patients, is associated with worse prognosis (64). Deciphering the immunosuppressive mechanisms of action of PMN shaped G-MDSCs will undoubtedly be relevant for understanding their origin and nature (47, 52, 58). Of relevance, LDGs with similar surface phenotype as G-MDSCs are isolated from patients with autoimmune disorders, with the important difference that these cells are pro-inflammatory (41). As for all MDSCs, only cells with a potent immunosuppressive capacity may be defined as MDSCs.

The immature neutrophils, produced as a response to tumor-induced stress and secreted colony stimulating factors GM-CSF, G-CSF, and M-CSF (5–10), could represent unique immature G-MDSC subpopulations. Their mechanisms of action, and also their capacity to differentiate into PMN shaped G-MDSCs, or neutrophils, will be important to delineate. An interesting and important issue for the future, is whether treating cancer patients with G-CSF for neutropenia, could affect the patients negatively in terms of G-MDSC enrichment, or not (65).

## Human G-MDSCs in Tumors

In mice, tumor infiltrating G-MDSCs are classically defined by the Ly6G marker. In humans, an equivalent marker has not yet been defined, since many candidate markers (e.g., CD15 or CD66b) are expressed on immature as well as mature neutrophils (Table 1). Indeed, immature and mature neutrophils are found both in the circulation of cancer patients (66, 67), and in human tumors (58, 68, 69), probably at varying density depending on tumor type and stage (58, 66–69). Of relevance, observations concerning migration and accumulation of immature and mature neutrophils in tumors have been made, where immature neutrophils have a reduced migratory capacity, but may still be able to accumulate at metastatic sites (40, 70, 71). Immature neutrophils have also been shown to survive longer in tumors (57). The immature and mature neutrophils in tumors may have different biology, as described above, and will thus affect disease severity differently (30). To define them as G-MDSCs, their immunosuppressive function is of outmost importance. The fact that a diversity of neutrophils is found in tumors have promoted researchers to define them as classical (N1) and alternative (N2) neutrophils (30, 53, 72). This has, however, only been experimentally shown in mice, and will still have to be determined in human tumors (41). Whether tumor infiltrating neutrophils derive from the immature neutrophil G-MDSCs as proposed in mice (66), or if they are reprogrammed or alternatively TGFβ activated N2 neutrophils (53, 73) will be interesting to follow. Until then, tumor infiltrating G-MDSCs may theoretically be grouped as immunosuppressive neutrophils (23). Novel methods as single cell RNA Seq, multiparameter immunofluorescence and mass cytometry by time of flight (CyTOF) will be valuable tools to decipher different subpopulations of tumor associated neutrophils (TANs) and G-MDSCs. Recently, using multidimensional imaging, LOX1^+^ tumor infiltrating G-MDSCs were reported to co-express the neutrophil activation marker MPO, and associate with immunosuppression and a worse prognosis (58, 63). For the future, TANs and G-MDSC may thus be targeted with similar, or vastly different therapeutic approaches.

## Human Peripheral Blood Mo-MDSCs

Mo-MDSCs are cells of the myeloid monocyte lineage, but with an HLA-DR^−/lo^ and co-receptor CD86^−/lo^ cell surface phenotype. They are potently immunosuppressive by soluble mediators like PGE_2_, IL10, TGFβ, and nitric oxide (NO), and enzymatic mediators like ARG1. As for all MDSCs, it is their immunosuppressive function that is key to defining them as Mo-MDSCs, and their functional mechanisms have been described in depth elsewhere (23, 39, 74–76). During leukocytosis, the emergency myelopoiesis is proposed to export immature myeloid cells into the circulation. This holds true for G-MDSCs, as discussed above, but not necessarily for Mo-MDSCs. The morphology of immature monocytic cells (monoblasts and promonocytes) are quite similar to the mature monocytes (77, 78). Also, the surface phenotype of immature monocytic cells (the CD11b^+^ promonocytes that could be accounted for being Mo-MDSCs), is very similar to mature monocytes (77, 78), including the HLA-DR^+^ phenotype, which is in contrast to Mo-MDSCs (Table 2). This makes it difficult to postulate that human Mo-MDSCs are immature cells.

**Table 2:** Surface phenotypes during monocyte differentiation, with expression levels of indicated markers in specific subpopulations indicated (77, 78).

	**GMP**	**Monoblast**	**Promonocyte**	**Monocyte**
CD33	+++	+++	+++	+++
CD34	+/++	+	–	–
CD36	–	–	++	+++
CD11b	–	–	++	+++
CD13	++	++	+/++	++/+++
CD14	–	–	+/++	+++
CD15	–	–	++	–/+
CD16	–	–	–	–/+
CD64	++	–	++	+++
HLA-DR	++	++	+++	++/+++

Mature monocytes come in different versions, with the most typical human monocytes being the classical (CD14^++^CD16^−^), non-classical (CD14^lo^CD16^+^), intermediate (CD14^+^CD16^+^) and as recently proposed the immunosuppressive Mo-MDSCs (CD14^+^HLA-DR^−/lo^) (79, 80). To date, proof showing that human Mo-MDSCs would be immature, or linked to an increased export of a specific subtype of immunosuppressive monocytes as proposed (79) is, however, still lacking. In contrast, current literature indicate that monocytes are plastic cells that can change surface phenotype and function depending on activation state and the local microenvironment (75, 79). Mo-MDSCs are enriched not only in patients with cancer, but also in patients with severe infections and inflammatory conditions (16). Mo-MDSCs have been proposed to be generated either through affected myelopoiesis, at the stage of activation, or both (61). Independent of which, STAT3 and NFκB inducing signals are required for their generation, and hence also for their key defining immunosuppressive function. STAT3 can be induced through soluble mediators like colony stimulating factors, IL10, IL6, or PGE_2_; and NFκB through pathogen recognition receptor (PRR) signaling triggered by pathogen associated molecular patterns (PAMPs) or sterile damage associated molecular patterns (DAMPs) (34, 61).

**Figure 3:**
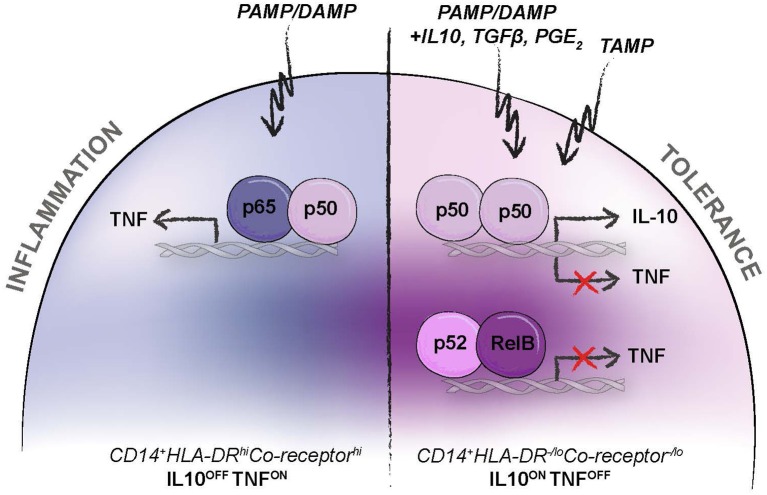
Reprogramming of systemic monocytes into anti-inflammatory monocytes. Human Mo-MDSCs have the same surface phenotype and function as anti-inflammatory monocytes that are reprogrammed by anti-inflammatory mediators (e.g., IL10, PGE_2_, TGFβ), pro-inflammatory pattern recognition receptor (PRR) ligands (e.g., PAMPs and DAMPs), or by endogenous anti-inflammatory PRR-ligands; tolerance associated molecular pattern (TAMPs), as previously suggested (87). This leads to transcriptional activation of *IL10* (*IL10* ON), inhibition of TNFα (*TNF* OFF), and acquisition of a CD14^+^HLA-DR^−/lo^Co-receptor^−/lo^ Mo-MDSC phenotype.

One example of disease-associated activation of monocytes, leading to cells with identical surface phenotype and immunosuppressive function as Mo-MDSCs, are the endotoxin tolerance reprogrammed monocytes present in the peripheral blood of sepsis patients (34, 81–85). Endotoxin tolerance is caused by pathogen and host response signals, with PAMPs as pathogen-induced TLR2/4-NFκB-signals and STAT3-inducing mediators like IL10, TGFβ, or PGE_2_ as host response signals (81, 86) (Figure 3). The combination of signal transduction lead to activation of alternative transcription factor NFκB complexes, consisting of homodimers of p50/p50 or heterodimers of p52/RelB, instead of the conventional p50/p65 heterodimers (34, 81–85). This leads to, transcriptional activation of *IL10* (*IL10* ON) and simultaneous inhibition of tumor necrosis factor alpha (TNFα) (*TNF* OFF) (Figure 3). When this happens, the mature peripheral blood monocytes acquire a CD14^+^HLA-DR^−/lo^Co-receptor^−/lo^ immunosuppressive and IL10 producing phenotype, suiting the Mo-MDSC criteria (81). Similar mechanisms also occur in a sterile environment, such as in a tumor or systemically in cancer patients, but with DAMPs rather than PAMPs as TLR-ligands (84, 85, 87–90). Downstream mediators of DAMP signaling, like PGE_2_, IL10, and NGF are already known to induce immunosuppressive myeloid cells (86, 87, 91, 92). One alternative explanation is the potential presence of unique anti-inflammatory endogenous PRR-ligands exclusively inducing transcriptional activation of *IL10* ON*/TNF* OFF (87, 93, 94). To differentiate between pro-inflammatory endogenous alarmins (DAMPs), we proposed to use the term tolerance-associated molecular patterns (TAMPs) for endogenous anti-inflammatory TLR-ligands (87). We have recently found a novel TLR4-ligand (Wnt5a) that is induced upon TLR4-signaling, and that functions as a TAMP activating the *IL10* ON/*TNF* OFF signal in primary human monocytes, resulting in Mo-MDSC-like cells by surface phenotype and function *in vitro*, a finding that was evolutionarily conserved in Drosophila (95), but not mice (87). In this context, a homeostatic feedback loop downstream of TLR2/4, would be able to regulate the acute pro-inflammatory response, as observed in sepsis. Other endogenous TLR-ligands have previously been observed to promote MDSC generation or to inhibit pro-inflammatory TLR signaling, like HMGB1 and S100B (89, 93, 94, 96). Interestingly, the MDSC marker S100A9 is a DAMP, binding to TLR4 (97).

Whether the plastic differentiation of mature monocytes into immunosuppressive monocytes is reversible or not, is not fully known. In patients with sepsis, the reprogrammed monocytes stay in their immunosuppressive state for up to 10 weeks with secondary infections and death as result, indicating that they may not be able to revert to a pro-inflammatory state again (16, 98). Opposite findings have, however, been presented for macrophages, where anti-inflammatory M2 macrophages where more plastic and differentiated into M1 macrophages upon stimulation with pro-inflammatory mediators (99, 100). It is undoubtfully so that the levels of peripheral blood Mo-MDSCs in cancer patients are associated with disease severity (76). Whether this is linked to an increased export of a certain subtype of immunosuppressive monocytes (79), or to reprogramming of circulating monocytes, is not clear and both may hold true. The latter (reversing reprogramming) may be more difficult to approach in a therapeutic setting.

## Human Mo-MDSCs in Tumors

When monocytes enter tissues, they are by definition differentiated into macrophages or monocyte-derived myeloid dendritic cells (mDCs). In an anti-inflammatory tumor microenvironment, this differentiation process is usually skewed into alternatively activated immunosuppressive macrophages of various kind, often exemplified by the simplistic M2 nomenclature, but also into mDCs (75, 101–103). Whether human myeloid cells like Mo-MDSCs also differentiate into macrophages in tumors is not fully known, although Mo-MDSCs have been shown to do so in mice (104). In humans, the hurdle lies with the difficulties to discriminate between the various human myeloid cell subsets with reliable results in tissues, and to be able to define their immunosuppressive function *in vivo*. In human tumors, various differentiation stages likely exist ranging from monocytes, Mo-MDSCs to tumor associated macrophages (TAMs), where markers are shared with those of Mo-MDSCs (74–76, 105). Also, isolation of myeloid populations from single cell suspensions of human tumors often generate subsets with less clear cell surface phenotypes as compared to myeloid cells from PBMCs. If the definition of Mo-MDSCs involves the CD14^+^HLA-DR^−/lo^ surface phenotype, it may thus be difficult to identify them in human tumors. If the definition of MDSCs within tumors are amended to describe them as immunosuppressive myeloid cells, tumor-associated MDSCs may be reconsidered and would include immunosuppressive macrophages as well. Still, the potential functional difference between tumor infiltrating Mo-MDSCs and alternatively activated macrophages is not elucidated in humans.

**Figure 4:**
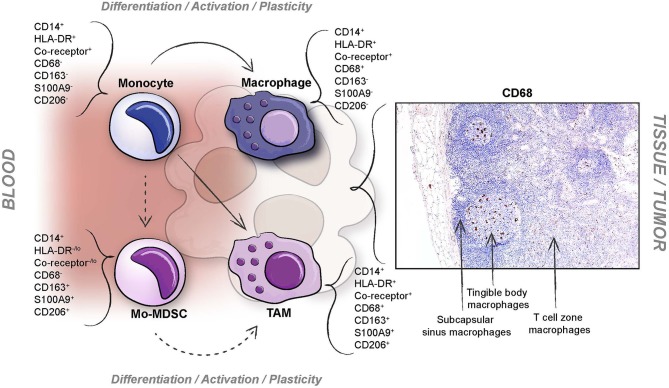
Difficulties when defining human Mo-MDSCs in tumors. Different issues ranging from plasticity of myeloid cells, technical problems concerning investigating multiparameter cell surface phenotypes in myeloid cells isolated from tissues, and definition of subpopulation markers; exemplified by the pan-macrophage marker CD68 which is variably expressed in different macrophage subpopulations (indicated here by a previously unpublished immunohistochemistry image representing staining of human resident lymph node macrophages with varying CD68 levels; using anti-CD68 clone KP1, diluted 1:1000, Dako, Glostrup, Denmark).

In mice, markers for Gr1 (Ly6C and Ly6G) are used to define MDSCs in tumors, and F4/80 to discriminate them from macrophages. Human Mo-MDSCs are still defined by a CD14^+^HLA-DR^−/lo^ surface phenotype. The immunosuppressive activity of Mo-MDSCs, which is critical for their definition, is for natural reasons more difficult, yet not less important, to assess in human tumors (74–76, 105). Furthermore, the dull CD14^+^HLA-DR^−/lo^ surface phenotype is often difficult to detect with accuracy using flow cytometry, resulting in a mixture of myeloid cells from tumor single cell suspensions. Since Mo-MDSCs by definition should have an immature myeloid cell phenotype, the macrophage maturation markers F4/80 in mice and CD68 in humans, should not be expressed (23). However, CD68 is expressed at various levels in macrophages, even when using established clinical pathology diagnostic markers (Figure 4), and it may therefore be difficult to discriminate Mo-MDSCs from TAMs by the use of conventional immunohistochemistry at present. Similar issues become clear when discussing the M2 TAM marker CD163. CD163 is expressed on a range of human anti-inflammatory myeloid cells producing IL10 (106–109) and is also upregulated by IL10, glucocorticoids and M-CSF (110). To complicate things, some tumor infiltrating CD163^+^ cells express low to negligible levels of the pan-macrophage marker CD68 (107, 111). Similarly, CD163 may be expressed on a subset of circulating anti-inflammatory CD14^+^HLA-DR^−/lo^ monocytes, Mo-MDSCs (112). It may therefore be of relevance to discuss whether CD163 can be expressed on tumor infiltrating Mo-MDSCs, or if all IL10 producing anti-inflammatory myeloid cells in tumors are M2 TAMs because of the CD163 expression. Recently, it was shown that primary human monocytes co-transplanted with xenografts upregulated both CD163 and nuclear S100A9 (113). Nuclear S100A9 has lately appeared as a human Mo-MDSC marker (36, 114). However, also the expression of S100A9, CD68, and CD163 in human myeloid cells seems to vary (115). Novel immunofluorescent multiparameter assays are being developed and a combination of markers like CD14, CD68, CD163, CD206, and S100A9 (108) is probably the best strategy to define Mo-MDSCs in human tumors, but their indispensable immunosuppressive function would need to be determined by other means. Single cell RNA Seq is a promising tool to provide us with more information on the functions of Mo-MDSCs in human tumors and tissues.

## Conclusions

**Figure 5:**
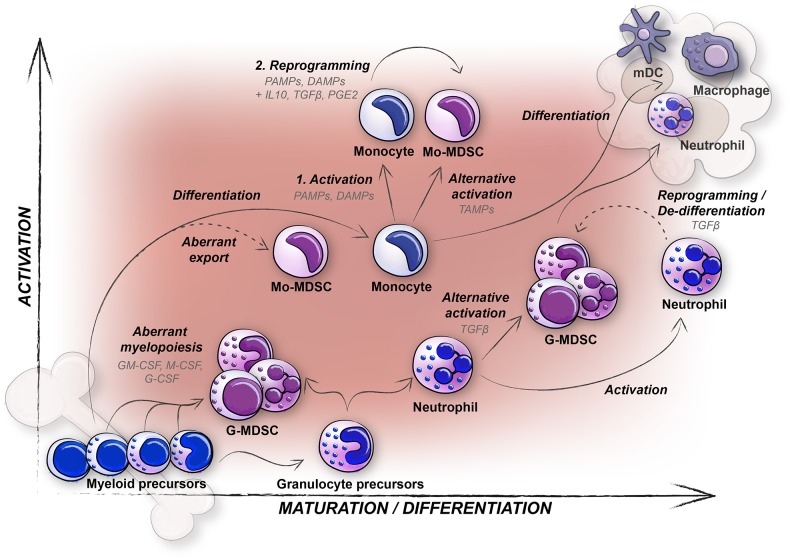
Concluding graphical summary featuring different hypotheses concerning the generation of human MDSCs (Mo-MDSCs and G-MDSCs), in relation to maturation and activation. Mo-MDSCs are predominantly generated from peripheral blood monocytes (solid arrows), and conceivably also through export of immature immunosuppressive monocytes although not yet proven in humans (dashed arrow). In contrast, G-MDSCs likely originate from aberrant myelopoiesis (immature neutrophil G-MDSCs) and alternative activation of mature neutrophils (mature neutrophil (PMN shaped) G-MDSCs, solid arrows). Whether also reprogramming or de-differentiation of mature neutrophils occur among G-MDSCs (dashed arrow) is yet to be determined in humans.

An imbalance in myelopoiesis reflects the biology of MDSCs well (Figure 5). All severe inflammatory conditions, including infection and cancer, affect MDSC generation. The challenge in terms of cancer is the strong interdependence between MDSCs and cancer cells. Cancer cells secrete factors that induce aberrant myelopoiesis (G-MDSC), as well as affect the myeloid cells already in circulation (Mo-MDSCs). The affected myeloid cells evolve in congruence with the tumor and metastasizing cells, and a constant feed-back loop is generated. In many cancer patients, myeloid cells are also unintentionally targeted during chemotherapy. To overcome this, patients are given G-CSF to boost myelopoiesis, leading to more MDSCs (65). An important challenge for our future knowledge on MDSCs is the translation from mouse to human MDSCs, where the essential immunosuppressive mediators like *ARG1* and *IL10* show an extreme polymorphism in humans but not mice (37, 116). Currently, drugs targeting all myeloid cells are being developed (75). It is important to stress that pro-inflammatory myeloid cells like lymph node resident macrophages and monocyte derived tumoricidal macrophages (M1 macrophages) and dendritic cells, are also needed for successful anti-tumor immune responses. Therefore, specific targeting of MDSC generating signals (e.g., STAT3), or the immunosuppressive MDSC specific functions (e.g., ARG1), should also be considered. With implementation of novel imaging and single cell analyses techniques, the origin of human MDSCs will undoubtfully be investigated in the near future. This will hopefully lead to answers on how to target human MDSCs as a therapeutic intervention in cancer patients. Such an intervention of the innate immunosuppressive arm, combined with the established check-point inhibition therapies targeting the adaptive immune response, could potentially offer a very potent therapeutic approach against cancer.

## Author Contributions

KL was responsible for writing the first draft. CB was responsible for the first figure drafts. KL and CB then together wrote, revised, and finalized the manuscript.

### Conflict of Interest

The authors declare that the research was conducted in the absence of any commercial or financial relationships that could be construed as a potential conflict of interest.
